# Open book technique for conjunctival tumors: a surgical approach to the fornix

**DOI:** 10.5935/0004-2749.20220025

**Published:** 2025-08-21

**Authors:** Jorge Agi, David I.T. Sia, Ezekiel Weis

**Affiliations:** 1 Department of Surgery, Faculty of Medicine and Dentistry, University of Calgary, Calgary, Canada; 2 Department of Ophthalmology and Visual Sciences, Faculty of Medicine and Dentistry, University of Alberta, Edmonton, Canada

**Keywords:** Fornix, brain, Lacrimal apparatus, Conjunctival neoplasm, Fornix, Aparelho lacrimal, Neoplasia da conjuntiva

## Abstract

Surgical management of large tumors involving the conjunctival fornix can be challenging, as exposure and clear margins may be difficult to achieve. In this case series, we report our initial experience with the open book technique in 4 patients. Through a canthotomy and cantholysis, this surgical approach provides a wide surgical field and facilitates fornix reconstruction post large tumor excision. In our series, one patient had a lateral canthus dehiscence.

## Abstract (pt)

O manejo cirúrgico de grandes tumores envolvendo o fórnice conjuntival pode ser desafiador, onde a exposição e as margens livres podem ser difíceis de serem alcançadas. Nesta série de casos, relatamos nossa experiência inicial com 4 pacientes usando a técnica de livro aberto. Através da cantotomia e da cantólise, esta abordagem cirúrgica fornece um amplo campo cirúrgico e também facilita a reconstrução do fórnice após a excisão de um tumor grande. Em nossa série, um paciente apresentou deiscência no canto lateral.

## INTRODUCTION

The conjunctiva is a thin and flexible mucous membrane that extends from the corneoscleral limbus (limbal conjunctiva) to the anterior surface of the globe (bulbar conjunctiva), down to the fornix (forniceal conjunctiva), onto the internal surface of the eyelid (palpebral conjunctiva), and up to the eyelid margin^(1)^.

A large series of 5,002 cases of conjunctival tumors showed the most common benign/premalignant lesions were nevus, primary acquired melanosis, and conjunctival intraepithelial neoplasia, while the most common malignant lesions were melanoma, squamous cell carcinoma, and lymphoma. The anatomical location most commonly involved is the limbal bulbar conjunctiva (47%), followed by the extralimbal bulbar conjunctiva (26%) and fornix (9%)^(1)^. The incidence of conjunctival lesions involving the fornix ranges from 4.2% to 9% in the literature^(1-3)^.

The surgical approach for lesions involving the conjunctival fornix can be straightforward for smaller tumors but is often challenging for larger lesions owing to the difficulty of exposure for clear margin excision and proper reconstruction. We describe in this report a surgical approach utilizing lateral canthotomy and cantholysis to improve the surgical exposure for resection and reconstruction of large conjunctival fornix tumors.

### Operative technique

Standardized surgical steps are followed prior to the procedure. The size of the lesion and nerve distributions involved dictate the choice of anesthesia, including general, retrobulbar, and subcutaneous local anesthesia. A subtenon or subconjunctival anesthesia is discouraged to avoid breaching the conjunctiva and inadvertent seeding to deeper layers or creating edema or hemorrhage, which makes the assessment of clinical margins difficult.

**Figure 1:**
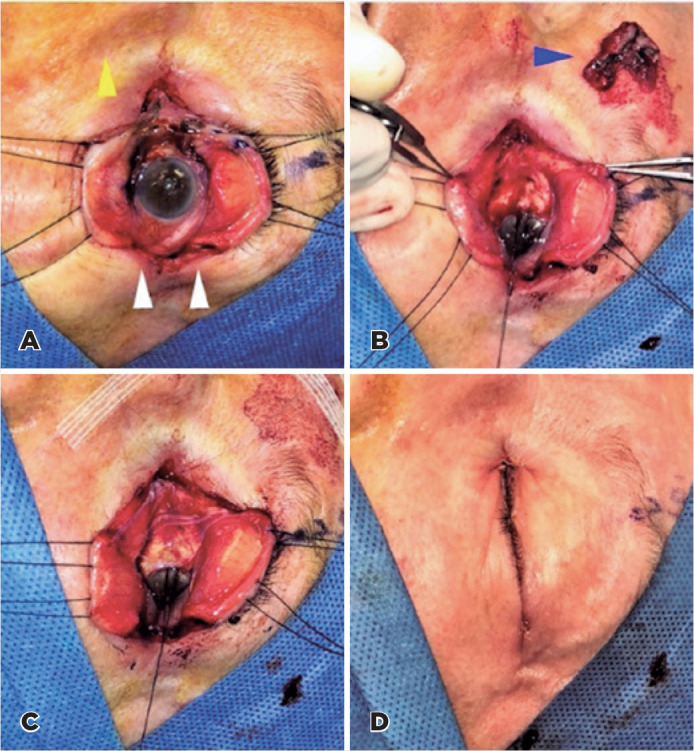
Color photograph illustrating the “open book” technique in Case 1. (A) Superior and inferior lateral canthotomy and cantholysis (white arrowheads) performed to allow traction sutures on the upper and lower lids to open the fornices like opening a book. The pigmented lesion involves the inferior conjunctival fornix, medial canthus, and upper and lower puncti (yellow arrowhead pointing to the nose). (B) A limbal traction suture is placed to abduct the globe. The pigmented lesion has been excised (blue arrowhead). (C) A double-layer amniotic membrane graft is placed over the excised area. The membrane is fixated with sutures and fibrin tissue glue. (D) Reconstruction of the lateral canthus with 4-0 Vicryl sutures followed by skin closure with a 6-0 plain gut.

Lateral canthotomy and inferior or superior cantholysis are performed depending on the location of the lesion. If both the upper and lower fornices are involved, a superior and inferior cantholysis can be performed. When performing an upper and lower cantholysis, the height of the location of the canthal tendon insertion on the lateral orbital rim is marked with a marking pen or cautery. Two 4-0 silk sutures are used to retract the upper and/or lower eyelids to expose the fornix (Figure 1A). The prior lateral canthotomy and cantholysis allow the eyelids to open up like a “book”; thereby, a flat surgical field can be achieved (Figure 1A).

A marking pen is used to mark the clinically evident tumor and the appropriate surgical margins around the tumor. A No. 15 blade and/or Wescott scissors are used to incise the conjunctiva/eyelid at the marked site. Bipolar cautery is applied to control bleeding, with attention not to cause damage by thermal injury during frozen assessment. To improve efficiency, the first step of the excision involves ensuring superficial margins 360° around the lesion. A frozen section is utilized to ensure complete resection with the creation of en face margins that are marked on the “true” outside margin. Careful and detailed labeling of the margins is critical owing to the large and often irregular shape of these lesions, which often require submitting multiple specimens for frozen section control. Once the margins are set, the lesion is excised with the “no touch” technique described by Shields et al.^(4)^ (Figure 1B). After the main lesion is excised, the base of the surgical bed can be reached, and a deep-frozen section margin is set. Repeat excisions of any positive margins are made until clear margins are achieved. For lesions that exhibit pagetoid spread, such as sebaceous cell carcinoma, perioperative map biopsies of the bulbar and palpebral conjunctiva are performed in advance for surgical planning.

Once a frozen section demonstrates clear margins, reconstruction is initiated. Owing to the large defects from advanced tumors, a double-layer amniotic membrane is used. The amniotic membrane graft is inserted with the stromal (basal layer) side placed on the open wound and fixated with 7-0 Vicryl sutures followed by application of fibrin tissue glue (Tisseel, Baxter, Illinois). A second layer of amniotic membrane is placed epithelial side down over the first layer, also completely covering the wound. This second layer is then fixated with 7-0 Vicryl sutures and fibrin tissue glue between the 2 amniotic layers (Tisseel, Baxter, Illinois; Figure 1C). To improve efficiency, if the amniotic membrane is large enough, an oversized first layer can be placed on the wound, with the basal layer down, followed by flipping the excess amniotic membrane onto the first layer of amniotic membrane. This results in a basal layer with the basement membrane down and a second layer with the epithelial side down. To reduce fornix symblepharon formation, a symblepharon ring is inserted during surgery and retained for approximately 1 week. Another technique is to use non-absorbable, non-braided fornix-deepening sutures in a horizontal mattress fashion, entering the fornix and tying on the cutaneous side (e.g., 3-0 nylon). Before fixating the eyelid back to the orbit, the tarsus is separated from the anterior lamellae by 2-3 mm, creating a small lateral tarsal strip. The incision for the lateral canthotomy and cantholysis is then closed with 2 simple interrupted 4-0 Vicryl sutures to Whitnall’s tubercle. Skin closure is completed with a simple interrupted 6-0 plain gut (Figure 1C), with the most medial suture just lateral to the upper eyelid lashes, allowing for a sharp V-shaped lateral canthus with no imbrication in most cases.

The complications of this approach include rounding of the lateral canthus, wound dehiscence, and longer healing time due to the extra incision and reconstruction. In advanced conjunctival tumors involving the fornix, we believe that the surgical advantages related to the improved access outweigh the risks.

## CASE REPORT

An 81-year-old woman (Patient 1; Table 1) was referred for evaluation of a pigmented lesion involving the left inferior fornix. This lesion was initially noticed by the patient 8 months before. In the last 2 months, she had noticed a rapid growth of the lesion. On clinical examination, a large pigmented lesion involving the left inferior conjunctival fornix, medial canthus, and upper and lower puncti was found. No abnormal lymph nodes were detected on palpation. The visual acuity was 20/25 OU, and extra-ocular movements were preserved. The patient underwent resection using the “open book” technique as described (Figure 1), and the canaliculi were reconstructed with Crawford tube insertion. Pathological assessment revealed a conjunctival melanoma with clear surgical margins. No signs of recurrence were found at 6-month follow-up.

**Table 1:** Cases of conjunctival fornix tumors excised with the “open book” technique

	Patient 1	Patient 2	Patient 3	Patient 4
Age, years	81	53	79	81
Sex	Female	Male	Female	Female
Location of lesion	Inferior fornix	Inferior fornix	Inferior palpebral, forniceal, and bulbar conjunctiva	Inferior fornix
Histological	Conjunctival	Squamous	Sebaceous	Squamous cell
diagnosis	melanoma	cell carcinoma	cell carcinoma	carcinoma in situ
Margin clearance	Clear	Clear	Clear	Clear
Complications	None	Lateral canthus dehiscence	None	None
Recurrence	No	No	No	No
Follow-up duration	6 months	32 months	10 months	26 months

Details of three further patients who underwent excision biopsy of large conjunctival fornix tumors with the “open book” technique is summarized in table 1.

## DISCUSSION

The “no touch” technique proposed by Shields et al.^(4)^ is a widely used surgical approach for excision of conjunctival tumors. However, using this technique on advanced forniceal and palpebral conjunctival tumors is often challenging owing to the difficult exposure of the fornix. This may complicate the assessment of clinical and frozen section margins and the subsequent reconstruction. The “open book” technique described herein is simply a combination of lateral canthotomy and cantholysis to improve access and visualization with the “no touch” technique for advanced conjunctival tumors involving the fornix. This approach represents the first step in the swinging eyelid technique used for accessing the orbit^(5,6)^. In our experience, it is a quick and efficient technique for improving visualization and aids tissue manipulation, as the fornix is laid flat for excision and subsequent reconstruction. This is especially pertinent when amniotic membrane grafts are used after large excisions involving the fornix. The time to perform these additional steps and the associated complications are similar to the well-described surgical steps in lateral canthotomy and cantholysis.

This technique is not appropriate for tumors involving the lateral canthus for three main reasons as follows: First, for lesions of the lateral canthus, if lateral canthotomy and cantholysis are performed, the surgeon is likely incise into the tumor, which potentially leads to seeding of tumor cells. Second, incising into the tumor eliminates the possibility of an en bloc excision, the ideal technique. Finally, en bloc excisions of lateral canthal lesions often require resection of the lateral canthus, which allows for excellent access to the fornix, making the canthotomy and cantholysis step redundant and unnecessary. In addition, for small tumors that can be easily excised and reconstructed without a canthotomy/ cantholysis, this technique can be avoided, thereby reducing surgical time, recovery time, and risks associated with this extra step.

To the best of our knowledge, the use of lateral canthotomy and cantholysis for resection and reconstruction of conjunctival fornix tumors has not been described. We found this technique useful for improving surgical exposure for achieving complete resection and subsequent reconstruction of large conjunctival fornix tumors.

## References

[1] Shields CL, Alset AE, Boal NS, Casey MG, Knapp AN, Sugarman JA (2017). Conjunctival tumors in 5002 cases. comparative analysis of benign
versus malignant counterparts. The 2016 James D. Allen
Lecture. Am J Ophthalmol.

[2] Mondal SK, Nag DR, Bandyopadhyay R, Adhikari A, Mukhopadhyay S. (2012). Conjunctival biopsies and ophthalmic lesions: A histopathologic
study in eastern India. J Res Med Sci.

[3] García Onrubia L, Pacheco-Callirgos GE, Portero-Benito A, García-Álvarez C, Carreño Salas E, Muñoz-Moreno MF (2020). Spectrum of conjunctival tumours in a Spanish series: A review of
462 cases. Eur J Ophthalmol.

[4] Shields JA, Shields CL, De Potter P. (1997). Surgical management of conjunctival tumors. The 1994 Lynn B.
McMahan Lecture. Arch Ophthalmol (Chicago, Ill 1960).

[5] McCord CD Jr (1981). Orbital decompression for Graves’ disease. Exposure through
lateral canthal and inferior fornix incision. Ophthalmology.

[6] Lyons CJ, Rootman J. (1994). Orbital decompression for disfiguring exophthalmos in thyroid
orbitopathy. Ophthalmology.

